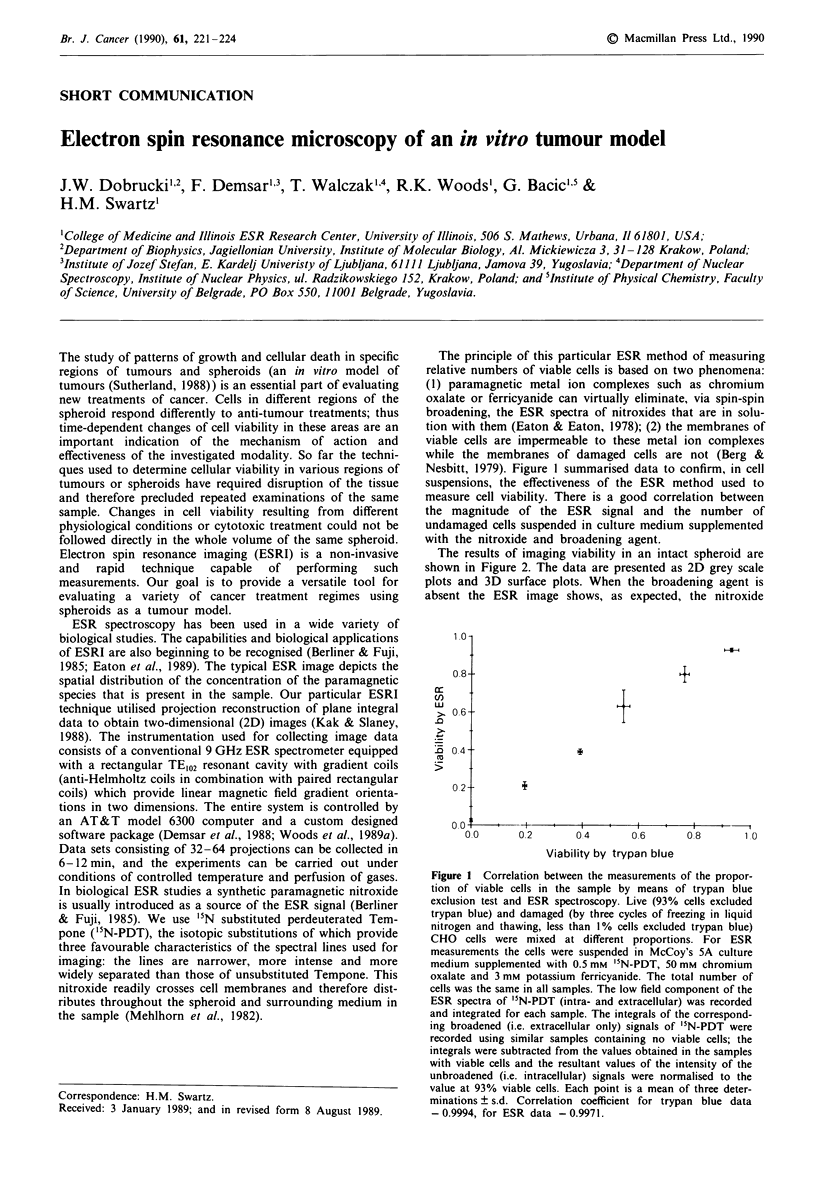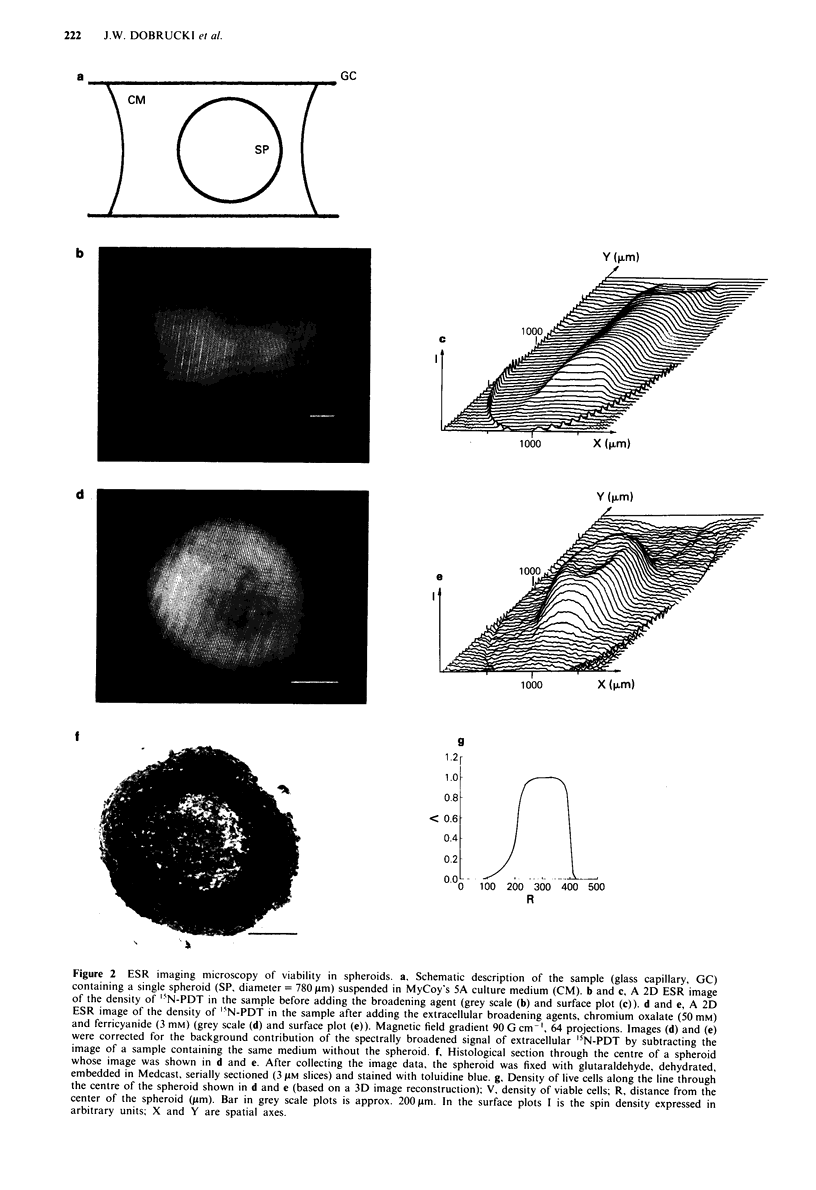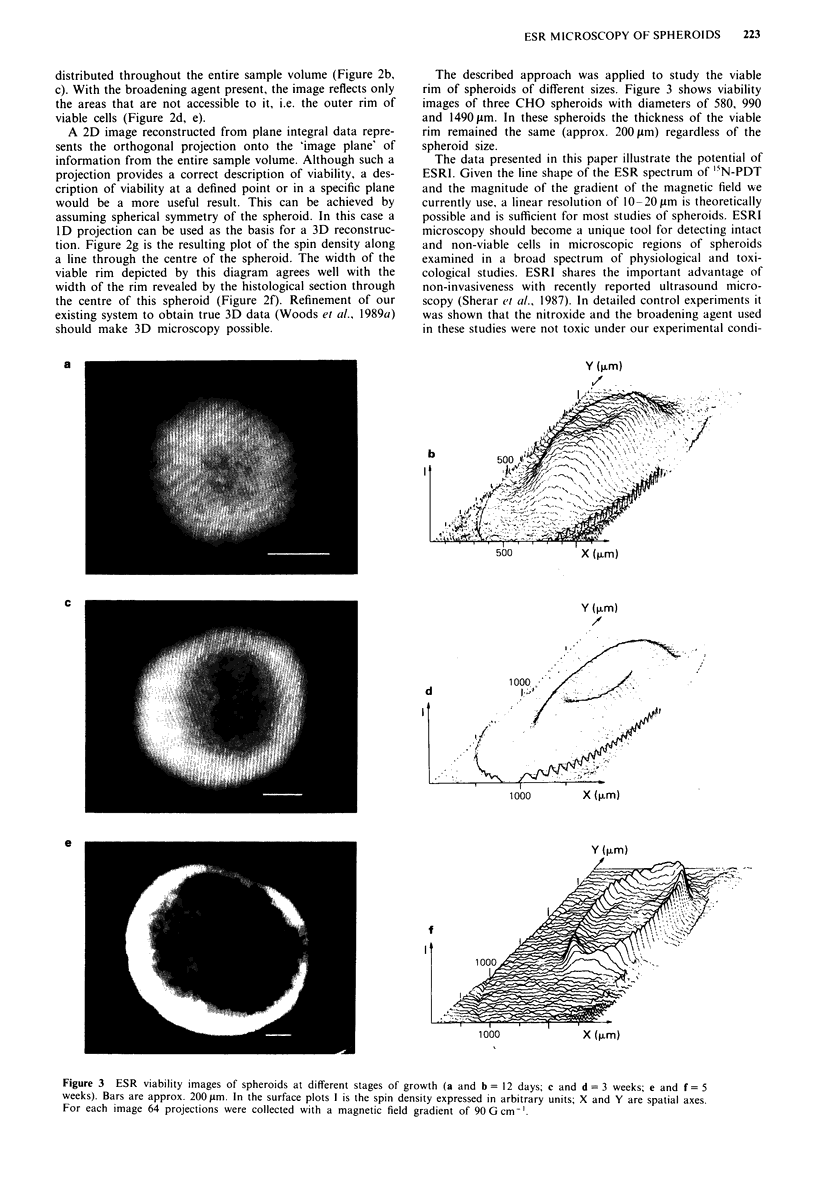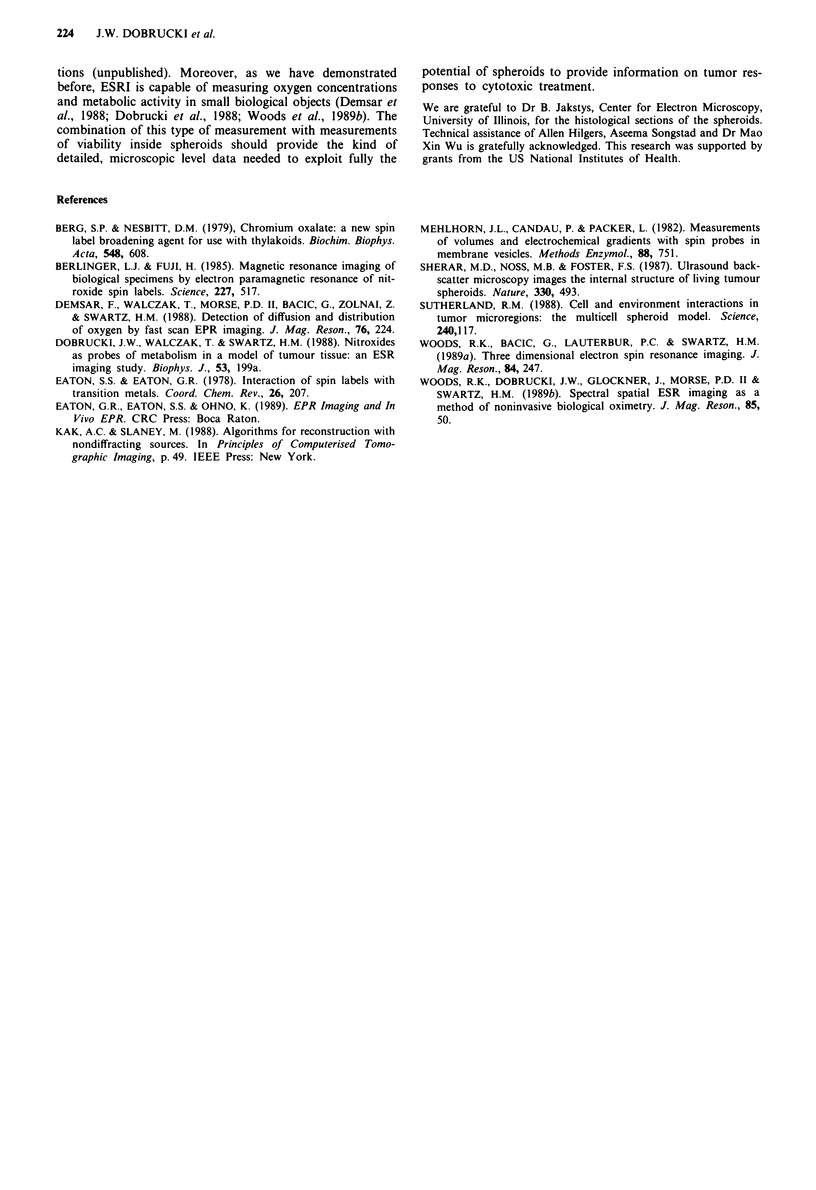# Electron spin resonance microscopy of an in vitro tumour model.

**DOI:** 10.1038/bjc.1990.41

**Published:** 1990-02

**Authors:** J. W. Dobrucki, F. Demsar, T. Walczak, R. K. Woods, G. Bacic, H. M. Swartz

**Affiliations:** College of Medicine, University of Illinois, Urbana 61801.

## Abstract

**Images:**


Br. J. Cancer (1990), 61, 221-224 © Macmillan Press Ltd., 1990

SHORT COMMUNICATION

### Electron spin resonance microscopy of an *in vitro* tumour model

J.W. Dobrucki[1,2], F. Demsar[1,3], T. Walczak[1,4], R.K. Woods[1], G. Bacic[1,5] & H.M. Swartz[1]

[1]*College of Medicine and Illinois ESR Research Center, University of Illinois, 506 S. Mathews, Urbana, Il 61801, USA;* [2]*Department of Biophysics, Jagiellonian University, Institute of Molecular Biology, Al. Mickiewicza 3, 31-128 Krakow, Poland;* [3]*Institute of Jozef Stefan, E. Kardelj Univeristy of Ljubljana, 61111 Ljubljana, Jamova 39, Yugoslavia;* [4]*Department of Nuclear Spectroscopy, Institute of Nuclear Physics, ul. Radzikowskiego 152, Krakow, Poland; and* [5]*Institute of Physical Chemistry, Faculty of Science, University of Belgrade, PO Box 550, 11001 Belgrade, Yugoslavia.*

The study of patterns of growth and cellular death in specific regions of tumours and spheroids (an *in vitro* model of tumours (Sutherland, 1988)) is an essential part of evaluating new treatments of cancer. Cells in different regions of the spheroid respond differently to anti-tumour treatments; thus time-dependent changes of cell viability in these areas are an important indication of the mechanism of action and effectiveness of the investigated modality. So far the techniques used to determine cellular viability in various regions of tumours or spheroids have required disruption of the tissue and therefore precluded repeated examinations of the same sample. Changes in cell viability resulting from different physiological conditions or cytotoxic treatment could not be followed directly in the whole volume of the same spheroid. Electron spin resonance imaging (ESRI) is a non-invasive and rapid technique capable of performing such measurements. Our goal is to provide a versatile tool for evaluating a variety of cancer treatment regimes using spheroids as a tumour model.

ESR spectroscopy has been used in a wide variety of biological studies. The capabilities and biological applications of ESRI are also beginning to be recognised (Berliner & Fuji, 1985; Eaton *et al.*, 1989). The typical ESR image depicts the spatial distribution of the concentration of the paramagnetic species that is present in the sample. Our particular ESRI technique utilised projection reconstruction of plane integral data to obtain two-dimensional (2D) images (Kak & Slaney, 1988). The instrumentation used for collecting image data consists of a conventional 9 GHz ESR spectrometer equipped with a rectangular $TE_{102}$ resonant cavity with gradient coils (anti-Helmholtz coils in combination with paired rectangular coils) which provide linear magnetic field gradient orientations in two dimensions. The entire system is controlled by an AT&T model 6300 computer and a custom designed software package (Demsar *et al.*, 1988; Woods *et al.*, 1989*a*). Data sets consisting of 32-64 projections can be collected in 6-12 min, and the experiments can be carried out under conditions of controlled temperature and perfusion of gases. In biological ESR studies a synthetic paramagnetic nitroxide is usually introduced as a source of the ESR signal (Berliner & Fuji, 1985). We use $^{15}N$ substituted perdeuterated Tempone ($^{15}N$-PDT), the isotopic substitutions of which provide three favourable characteristics of the spectral lines used for imaging: the lines are narrower, more intense and more widely separated than those of unsubstituted Tempone. This nitroxide readily crosses cell membranes and therefore distributes throughout the spheroid and surrounding medium in the sample (Mehlhorn *et al.*, 1982).

Correspondence: H.M. Swartz.
Received: 3 January 1989; and in revised form 8 August 1989.

The principle of this particular ESR method of measuring relative numbers of viable cells is based on two phenomena: (1) paramagnetic metal ion complexes such as chromium oxalate or ferricyanide can virtually eliminate, via spin-spin broadening, the ESR spectra of nitroxides that are in solution with them (Eaton & Eaton, 1978); (2) the membranes of viable cells are impermeable to these metal ion complexes while the membranes of damaged cells are not (Berg & Nesbitt, 1979). Figure 1 summarised data to confirm, in cell suspensions, the effectiveness of the ESR method used to measure cell viability. There is a good correlation between the magnitude of the ESR signal and the number of undamaged cells suspended in culture medium supplemented with the nitroxide and broadening agent.

The results of imaging viability in an intact spheroid are shown in Figure 2. The data are presented as 2D grey scale plots and 3D surface plots. When the broadening agent is absent the ESR image shows, as expected, the nitroxide

**Figure 1** Correlation between the measurements of the proportion of viable cells in the sample by means of trypan blue exclusion test and ESR spectroscopy. Live (93% cells excluded trypan blue) and damaged (by three cycles of freezing in liquid nitrogen and thawing, less than 1% cells excluded trypan blue) CHO cells were mixed at different proportions. For ESR measurements the cells were suspended in McCoy's 5A culture medium supplemented with 0.5 mM $^{15}N$-PDT, 50 mM chromium oxalate and 3 mM potassium ferricyanide. The total number of cells was the same in all samples. The low field component of the ESR spectra of $^{15}N$-PDT (intra- and extracellular) was recorded and integrated for each sample. The integrals of the corresponding broadened (i.e. extracellular only) signals of $^{15}N$-PDT were recorded using similar samples containing no viable cells; the integrals were subtracted from the values obtained in the samples with viable cells and the resultant values of the intensity of the unbroadened (i.e. intracellular) signals were normalised to the value at 93% viable cells. Each point is a mean of three determinations ± s.d. Correlation coefficient for trypan blue data - 0.9994, for ESR data - 0.9971.

**Figure 2** ESR imaging microscopy of viability in spheroids. **a**, Schematic description of the sample (glass capillary, GC) containing a single spheroid (SP, diameter = 780 μm) suspended in MyCoy's 5A culture medium (CM). **b** and **c**, A 2D ESR image of the density of $^{15}$N-PDT in the sample before adding the broadening agent (grey scale (**b**) and surface plot (**c**)). **d** and **e**, A 2D ESR image of the density of $^{15}$N-PDT in the sample after adding the extracellular broadening agents, chromium oxalate (50 mM) and ferricyanide (3 mM) (grey scale (**d**) and surface plot (**e**)). Magnetic field gradient 90 G cm$^{-1}$, 64 projections. Images (**d**) and (**e**) were corrected for the background contribution of the spectrally broadened signal of extracellular $^{15}$N-PDT by subtracting the image of a sample containing the same medium without the spheroid. **f**, Histological section through the centre of a spheroid whose image was shown in **d** and **e**. After collecting the image data, the spheroid was fixed with glutaraldehyde, dehydrated, embedded in Medcast, serially sectioned (3 μM slices) and stained with toluidine blue. **g**, Density of live cells along the line through the centre of the spheroid shown in **d** and **e** (based on a 3D image reconstruction); V, density of viable cells; R, distance from the center of the spheroid (μm). Bar in grey scale plots is approx. 200 μm. In the surface plots I is the spin density expressed in arbitrary units; X and Y are spatial axes.

distributed throughout the entire sample volume (Figure 2b, c). With the broadening agent present, the image reflects only the areas that are not accessible to it, i.e. the outer rim of viable cells (Figure 2d, e).

A 2D image reconstructed from plane integral data represents the orthogonal projection onto the 'image plane' of information from the entire sample volume. Although such a projection provides a correct description of viability, a description of viability at a defined point or in a specific plane would be a more useful result. This can be achieved by assuming spherical symmetry of the spheroid. In this case a 1D projection can be used as the basis for a 3D reconstruction. Figure 2g is the resulting plot of the spin density along a line through the centre of the spheroid. The width of the viable rim depicted by this diagram agrees well with the width of the rim revealed by the histological section through the centre of this spheroid (Figure 2f). Refinement of our existing system to obtain true 3D data (Woods *et al.*, 1989*a*) should make 3D microscopy possible.

The described approach was applied to study the viable rim of spheroids of different sizes. Figure 3 shows viability images of three CHO spheroids with diameters of 580, 990 and 1490 μm. In these spheroids the thickness of the viable rim remained the same (approx. 200 μm) regardless of the spheroid size.

The data presented in this paper illustrate the potential of ESRI. Given the line shape of the ESR spectrum of $^{15}$N-PDT and the magnitude of the gradient of the magnetic field we currently use, a linear resolution of 10–20 μm is theoretically possible and is sufficient for most studies of spheroids. ESRI microscopy should become a unique tool for detecting intact and non-viable cells in microscopic regions of spheroids examined in a broad spectrum of physiological and toxicological studies. ESRI shares the important advantage of non-invasiveness with recently reported ultrasound microscopy (Sherar *et al.*, 1987). In detailed control experiments it was shown that the nitroxide and the broadening agent used in these studies were not toxic under our experimental condi-

**Figure 3** ESR viability images of spheroids at different stages of growth (**a** and **b** = 12 days; **c** and **d** = 3 weeks; **e** and **f** = 5 weeks). Bars are approx. 200 μm. In the surface plots I is the spin density expressed in arbitrary units; X and Y are spatial axes. For each image 64 projections were collected with a magnetic field gradient of 90 G cm$^{-1}$.

tions (unpublished). Moreover, as we have demonstrated before, ESRI is capable of measuring oxygen concentrations and metabolic activity in small biological objects (Demsar *et al.*, 1988; Dobrucki *et al.*, 1988; Woods *et al.*, 1989*b*). The combination of this type of measurement with measurements of viability inside spheroids should provide the kind of detailed, microscopic level data needed to exploit fully the potential of spheroids to provide information on tumor responses to cytotoxic treatment.

We are grateful to Dr B. Jakstys, Center for Electron Microscopy, University of Illinois, for the histological sections of the spheroids. Technical assistance of Allen Hilgers, Aseema Songstad and Dr Mao Xin Wu is gratefully acknowledged. This research was supported by grants from the US National Institutes of Health.

### References

BERG, S.P. & NESBITT, D.M. (1979), Chromium oxalate: a new spin label broadening agent for use with thylakoids. *Biochim. Biophys. Acta*, **548**, 608.

BERLINGER, L.J. & FUJI, H. (1985). Magnetic resonance imaging of biological specimens by electron paramagnetic resonance of nitroxide spin labels. *Science*, **227**, 517.

DEMSAR, F., WALCZAK, T., MORSE, P.D. II, BACIC, G., ZOLNAI, Z. & SWARTZ, H.M. (1988). Detection of diffusion and distribution of oxygen by fast scan EPR imaging. *J. Mag. Reson.*, **76**, 224.

DOBRUCKI, J.W., WALCZAK, T. & SWARTZ, H.M. (1988). Nitroxides as probes of metabolism in a model of tumour tissue: an ESR imaging study. *Biophys. J.*, **53**, 199a.

EATON, S.S. & EATON, G.R. (1978). Interaction of spin labels with transition metals. *Coord. Chem. Rev.*, **26**, 207.

EATON, G.R., EATON, S.S. & OHNO, K. (1989). *EPR Imaging and In Vivo EPR*. CRC Press: Boca Raton.

KAK, A.C. & SLANEY, M. (1988). Algorithms for reconstruction with nondiffracting sources. In *Principles of Computerised Tomographic Imaging*, p. 49. IEEE Press: New York.

MEHLHORN, J.L., CANDAU, P. & PACKER, L. (1982). Measurements of volumes and electrochemical gradients with spin probes in membrane vesicles. *Methods Enzymol.*, **88**, 751.

SHERAR, M.D., NOSS, M.B. & FOSTER, F.S. (1987). Ulrasound backscatter microscopy images the internal structure of living tumour spheroids. *Nature*, **330**, 493.

SUTHERLAND, R.M. (1988). Cell and environment interactions in tumor microregions: the multicell spheroid model. *Science*, **240**,117.

WOODS, R.K., BACIC, G., LAUTERBUR, P.C. & SWARTZ, H.M. (1989*a*). Three dimensional electron spin resonance imaging. *J. Mag. Reson.*, **84**, 247.

WOODS, R.K., DOBRUCKI, J.W., GLOCKNER, J., MORSE, P.D. II & SWARTZ, H.M. (1989*b*). Spectral spatial ESR imaging as a method of noninvasive biological oximetry. *J. Mag. Reson.*, **85**, 50.